# A Paired Database of Predicted and Experimental Protein Peptide Binding Information

**DOI:** 10.1038/s41597-025-05754-7

**Published:** 2025-08-20

**Authors:** Jazmine A. Torres, Chris A. Kieslich, Robert J. Pantazes

**Affiliations:** 1 Department of Chemical Engineering, Auburn University, Auburn, AL 36849-5127 USA; 2 Wallace H. Coulter Department of Biomedical Engineering, Georgia Institute of Technology, Atlanta, GA 30332 USA

**Keywords:** Computational models, Protein function predictions, Proteins, Peptides

## Abstract

Peptides are important biomolecules, and their interactions with proteins make them useful in sensing and therapeutic applications. Computational peptide design methods can benefit from high-quality peptide-protein structures paired with thermodynamic data. The Predicted and Experimental Peptide Binding Information (PEPBI) database provides 329 predicted peptide-protein complexes, each based on an experimentally determined structure, with corresponding experimental measurements of changes in Gibbs free energy, enthalpy, and entropy. For each complex, 40 properties calculated using Rosetta’s Interface Analyzer are included. Complexes were selected for inclusion in PEPBI using eight stringent structural criteria, including peptide length (5–20 residues), structure resolution (≤2.0 Å), less than 30% sequence identity between complexes, and having a corresponding unbound protein structure in the Protein Data Bank with at least 90% sequence identity to the bound form with minimal changes in the binding pocket. PEPBI is expected to be of use for the development of computational methods for peptide design with desired binding properties to protein targets.

## Background & Summary

Like proteins, peptides are biomolecules composed of amino acids. Although the exact definition of what constitutes a peptide varies in different literature reports^1–3^, they are commonly understood to be short sequences without defined tertiary structures. Peptides play crucial roles in biological systems by regulating a variety of cellular processes through interactions with proteins^4,5^. Peptide-protein complexes are estimated to make up between 15 to 40% of all intracellular interactions^5,6^, with peptides filling diverse functions including structural elements^7^, hormones^8^, neurotransmitters^9^, growth factors^10^, etc. Based on their wide range of significant naturally occurring roles, humanity has leveraged peptides for a number of critical applications, including as diagnostic tools^11^, therapeutics^8,11^, and drug delivery agents^7^.

Understanding the structures and thermodynamic properties of how peptides bind to proteins can aid the successful development of peptides for these applications. X-ray crystallography^12^, cryogenic-electron microscopy (Cryo-EM)^13^, and nuclear magnetic resonance (NMR)^14^ have been used to study the structures of protein-peptide complexes, while methods such as surface plasmon resonance (SPR)^15^ and isothermal titration calorimetry (ITC)^16,17^ have been used to assess the thermodynamic properties of their interactions. However, these experimental methods are often resource-intensive and impractical in terms of scalability, which pose challenges in thoroughly exploring the sequence landscape, structural diversity, and interaction networks of peptides. To address the constraints of experimental approaches, computational methods have become an integral part of peptide research and design pipelines. Computational tools such as force fields^18,19^, molecular dynamics (MD) simulations^18,19^, molecular docking^6,20^, machine learning^20,21^, and many others^22–33^ have all been used for peptide structure prediction and design efforts.

Like other thermodynamic processes, spontaneous peptide-protein binding events have negative changes in Gibbs free energy (ΔG), which is itself a function of changes in enthalpy (ΔH) and entropy (ΔS). Computational methods, especially ones based on increasingly popular machine learning methods^34–37^, require experimental training data during their development. For peptide-protein systems, this should include both structural data and corresponding thermodynamic information, with data on ΔS expected to be of particular importance. As defined here, peptides lack tertiary structure and are therefore disordered in solution. This means they lose many degrees of freedom upon protein binding and have correspondingly large entropic penalties. While there are many literature reports of structural and thermodynamic characterizations of protein-peptide complexes, including databases of one or the other^38–40^, we were unable to locate a prior report listing complexes with both A) known structures and B) direct reports of ΔG, ΔH, and ΔS or sufficient thermodynamic information to calculate these properties. Therefore, we have curated the Predicted and Experimental Peptide Binding Information (PEPBI) database, which contains 329 peptide-protein complexes with the aforementioned structural and thermodynamic data and corresponding computational predictions of binding properties.

**Fig. 1:**
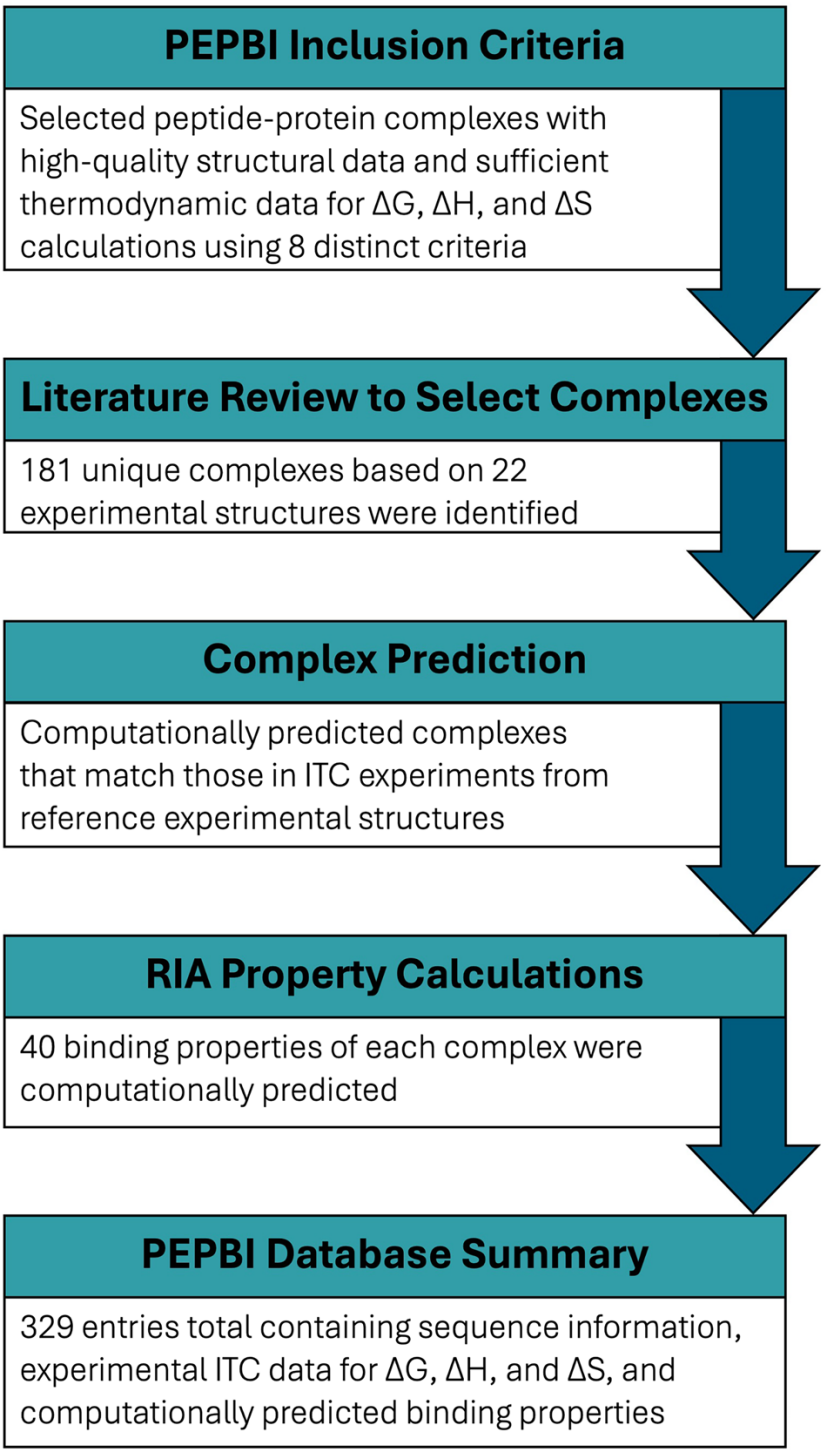
The Workflow Used for the Development of PEPBI. PEPBI was curated using a five-step process. The first step was the definition of the criteria for complexes to be included. In the next step, a literature review was conducted to identify the peptide-protein complexes that should be part of PEPBI. Once they were known, complexes that exactly match the proteins and peptides used in the ITC experiments were computationally predicted. This was followed by calculating properties of those complexes using the Rosetta Interface Analyzer. Ultimately, this yielded the PEPBI database of 329 peptide-protein complexes with matched thermodynamic data.

As depicted in Fig. 1, PEPBI was constructed through a literature review process whereby protein-peptide complexes with sufficient structural and thermodynamic data were identified. The required structural information was an experimentally determined complex of the protein and peptide or a sufficiently related system (e.g., a peptide that had several amino acid mutations from the reported peptide). The thermodynamic information that was needed was either a direct report of the three thermodynamic variables (ΔG, ΔH, and ΔS) or sufficient information to calculate them from the available data. Once complexes were identified, missing properties were calculated (e.g., ΔG from a dissociation constant (k_D_) and temperature) or predicted (e.g., portions of protein structures that were experimentally unresolved were predicted with RoseTTAFold^35^). Finally, the predicted complexes were energetically minimized and computational binding properties were calculated. The data in PEPBI is expected to be of use for training computational models for designing protein-peptide binding complexes.

## Methods

PepSet is a previously reported database of peptide-protein complexes^6^. It was itself extracted from PepBDB^41^, which is a database of every peptide-protein complex from the Protein Data Bank (PDB)^41^ with peptides of 50 or fewer amino acids. Six criteria were used to select complexes for inclusion in PepSet, each of which is also relevant for PEPBI. The first PepSet criteria was that peptides had to be between 5 and 20 amino acids long^6^. The exact definition of what constitutes a peptide can vary^1–3^, but limiting the number of amino acids ensures that the included peptides lack tertiary structure. The next PepSet criteria was that the experimentally determined structures had resolutions ≤ 2.0 Å. Using this restriction ensures that only high-quality experimental structures are included in PEPBI and provides confidence that the intermolecular interactions they contain are accurate. PepSet’s third criteria was that the peptides be composed of only the 20 common amino acids^6^. This restriction is essential for PEPBI because PEPBI includes computational predictions of properties of the protein-peptide complexes made with the Rosetta force field^42^, for which the most validated parameterization is for the 20 canonical amino acids.

PepSet’s fourth criteria was that proteins of different complexes have sequence identities of <30%^6^, which is a common threshold for homology-based protein structure prediction^43^. Use of this cutoff helps ensure that PEPBI considers a diverse set of protein-peptide complexes. The fifth criteria to include a protein-peptide complex in PepSet was that a structure be available in the PDB of an unbound version of the protein with at least 90% sequence identity to the protein in the complex, and the sixth is that the root mean squared deviation (RMSD) of the backbone atoms of the protein’s amino acids within 10 Å of the peptide be ≤ 2.0 Å when compared to the unbound protein^6^. Although PEPBI does not include models of the unbound proteins, the final two PepSet requirements are of particular importance. The purpose motivating PEPBI’s development is to gather experimental structural and thermodynamic data for developing computational models of protein-peptide binding. The relevant thermodynamic properties measure changes between the bound and unbound states, so the existence of experimental structural data for the unbound proteins may be important to the successful development of those models. Structures of the unbound peptides cannot be included in PEPBI because they do not exist, so the choice was made to also not include the unbound protein structures in the database. However, their existence is an important constraint for PEPBI.

Overall, PepSet used a conservative set of requirements to identify all protein-peptide complexes from the PDB that have high-quality experimental data. As each of the requirements of PepSet is also relevant for PEPBI, the 185 protein-peptide complexes identified for PepSet were those considered for inclusion in PEPBI. In addition to the six PepSet criteria, there were two additional criteria for inclusion in PEPBI. The first was that the protein only contain the 20 common amino acids and not have any post-translational modifications, which is necessary for the computational calculations and is analogous to PepSet’s equivalent criteria for the peptides. The second requirement is that there be ITC data that directly reports or is sufficient to calculate ΔG, ΔH, and ΔS. After a literature review and contacting the corresponding author of every manuscript that reported only some of the necessary ITC data, 22 protein-peptide complexes were identified that met the full list of eight criteria necessary for inclusion in PEPBI.

The reports describing the 22 identified protein-peptide complexes also included ITC data for an additional 160 mutated complexes. All such complexes were also included in PEPBI, for a total of 181 unique peptide-protein complexes. In nine of these complexes, the peptides exceeded the PepSet length limit of 20 amino acids. PEPBI used this limit to ensure that the peptides are sufficiently short that they do not have tertiary structure. As each of these nine complexes was one where the peptide bound the protein in the same manner as a shorter peptide, it was determined that including them in PEPBI was appropriate.

The minimum ITC data for inclusion in PEPBI was an experimental report of T, ΔH, and either ΔG or k_D_. ΔG and k_D_ can be calculated from one another when T is known, and ΔS can be calculated when T, ΔG, and ΔH are known. In addition to the minimum thermodynamic data, experimentally reported values for the protein-peptide binding stoichiometry (N), TΔS, ΔS, and the standard deviations of all values were collected for inclusion in PEPBI.

Although there are experimental structures and ITC data for every protein-peptide complex, there were portions of the protein structures that needed to be corrected. These occurred due to undetermined regions in the structure determination experiments, protein sequence differences between the structural and ITC experiments (e.g., due to unremoved purification tags like poly-HIS), and mutations between peptides and/or proteins in ITC experiments for the same experimental structures. Therefore, computational methods were used to predict the structures of the protein-peptide complexes based on the experimental structures.

When proteins had gaps in their experimentally determined structures (i.e., cases where there were resolved amino acids both before and after the missing regions), the gaps were predicted using the built-in version of Modeller^44^, a tool for comparative structure modelling, in UCSF Chimera version 1.17.1^45^, a molecular visualization and analysis tool. In cases where there were five or fewer missing residues at an N- or C-termini of the protein, they were manually inserted using Chimera^45^. In cases where more than five terminal residues were missing, RoseTTAFold^35^ was used to predict the structure of the entire protein and the missing portion was computationally spliced onto the experimental structure. The different protocol was used to account for the possibility that longer missing fragments may have secondary structural features or intraprotein interactions that manual insertion cannot predict. When the experimental structures had extra residues on a terminus, they were deleted in Chimera^45^. Some complexes used protein homologues to the experimentally determined ones. In such cases, the homologue structure was predicted using RoseTTAFold^35^ and then superimposed on the original structure using Chimera^45^. Mutations of proteins were done using the swap amino acid tool of Chimera^45^.

When peptides were missing termini residues, they were predicted using Modeller^44^ when possible (i.e., when they are listed in the PDB files as missing residues) and manually inserted otherwise. All internal gaps in peptide sequences were predicted using Modeller^44^. RoseTTAFold^35^ could not be used to predict peptide sequences because it has a minimum sequence length of 26 amino acids. All peptide sequence changes, even ones involving changes to more than half of a peptide’s sequence, were done by mutating positions using Chimera’s swap amino acid tool^45^ and deleting and/or inserting termini residue as described.

Some experimental structures contain multiple copies of the protein-peptide complex. For example, PDB file 2IVZ^46^ contains four copies of the TolB protein in complex with the ColE9 peptide. In such cases, complexes were predicted for each individual subcomplex, as well as an additional complex using all copies of the protein and peptide. In the case of 2IVZ, this resulted in five total complexes being included in PEPBI: one for each of the four individual copies of the protein-peptide complex and one containing all four copies simultaneously. Doing so resulted in 329 total peptide-protein complexes being created for the 181 complexes with thermodynamic data. Table 1 lists the names of the protein-peptide complex binding groups, the PDB files containing their structural data, how many copies of the protein-peptide complex were in the original PDB file, the source of the ITC experimental data, how many variants were tested in the ITC experiments, and how many entries are included in PEPBI.

**Table 1:** PEPBI Database Summary.

Binding Groups	PDB Files	No. of Experimental Structure Copies	ITC Source	ITC Complexes Tested	PEPBI Entries
Fyn SH3 - P2L	1A0N^55^	1	^56^	1	1
NbSyn2 - α-synuclein	2X6M^57^	1	^12^	4	4
TolB - ColE9	2IVZ^46^	4	^58^	1	5
hRpn13 Pru Domain - hRpn2	6CO4^59^	1	^60^	2	2
DD Construct - Pro Peptides	4BTB^61^	1	^62^	2	2
GephE - GlyR β-subunit	4PD1^63^	1	^64^	2	2
ClpV-N - P-VipB	3ZRJ^65^	2	^66^	2	6
CaVβ2a - AID	5V2P^67^	1	^68^	3	3
APC - Amer1 Fragment	4YJE^69^	1	^70^	3	3
Tat_SF1 - SF3b1 ULM Fragment	6N3E^71^	1	^72^	3	3
CAPERα UHM - ULM Fragment	4OZ1^73^	1	^74^	3	6
SGT2-TPR - Ssa1 Fragment	5LYN^75^	2	^14^	3	9
PTPA - PP2A Catalytic Subunit	4NY3^76^	2	^77^	4	12
PHD-UHRF1 - H3	3SHB^78^	1	^79^	6	6
Pyk2-FAT - Leupaxin	4XEF^80^	2	^81^	4	20
	4XEK^82^				
Proflin IIa - VASP	2V8C^83^	1	^84^	3	3
Proflin IIa - mDia1	2V8F^85^	1	^84^	3	3
SOCS - hPar-4	2JK9^86^	1	^87^	3	3
SOCS - VASA	3F2O^88^	2	^87^	3	9
ATG8-like Protein - KBTBD6 AIM	4XC2^89^	1	^90^	3	3
ATG8-like Proteins - KBTBD7 AIM	4XC2^89^	1	^90^	6	6
SUMO1 - PIASX SIM	2LAS^91^	1	^92^	5	5
SUMO2 - PIASX SIM	2LAS^91^	1	^92^	6	6
SH3 STAC Fragment - CaV1.1	6B27^93^	6	^94^	5	35
α-adaptin - Motif Peptide	1W80^95^	1	^96^	27	27
β-adaptin - Motif Peptide	1W80^95^	1	^96^	5	5
TtSlyD - S2 Fragment	4ODL^97^	1	^98^	64	128
TtSlyD - S3 Fragment	4ODL^97^	1	^98^	2	2
TtSlyD - SlpA Linker	4ODL^97^	1	^98^	2	2
TtSlyD - T1 Fragment	4ODK^99^	1	^98^	1	1
TtSlyDΔIF - S2 Fragment	4ODP^100^	1	^98^	6	6
TtSlyDΔIF - S3 Fragment	4ODQ^101^	1	^98^	1	1

This summary includes the following for each binding group: **1**. PDB files used for structural data, **2**. the number of protein-peptide complex copies present in the PDB files, **3**. ITC data sources, **4**. the number of variants tested in ITC studies, and **5**. the number of data entries in PEPBI.

Each of the 329 complexes was subjected to the same model refinement and analysis process. First, they underwent an all-atom Chemistry at Harvard Molecular Mechanics (CHARMM) 36^47^ energy minimization using a harmonic force constant of 10 $\frac{{kcal}}{{mol}\cdot {\mathring{\rm A} }^{2}}$ on the N, Cα, and C backbone atoms and using the bond, angle, Urey-Bradley, dihedral angle, improper dihedral angle, electrostatics, and van der Waals energy terms. This was followed by an unrestrained, all-atom energy minimization using the Rosetta force field^42^. Finally, Rosetta’s Interface Analyzer (RIA) tool was used to predict 40 binding properties of the protein-peptide complexes^48^. They are listed in Table 2, and include binding energies, buried surface areas, shape complementarities, etc. Using CHARMM followed by Rosetta is consistent with prior works from our lab^49,50^ and is due to our observation that CHARMM can computationally correct clashes that cause Rosetta to fail, while Rosetta is both the most widely used protein design force field and RIA provides a more comprehensive report of protein properties than CHARMM.

**Table 2:** The RIA-calculated Properties.

RIA Property Name	Brief Description
total_score	Total energy for the complex
complex_normalized	Total energy for the complex divided by the total number of amino acids
dG_cross	Predicted binding energy using cross interface terms (not recommended for use)
dG_cross/dSASAx100	dG_cross divided by change in buried surface area
dG_separated	Predicted binding energy
dG_separated/dSASAx100	dG_separated divided by change in buried surface area.
dSASA_hphobic	Change in hydrophobic solvent accessible surface area.
dSASA_int	Change in total solvent accessible surface area
dSASA_polar	Change in polar solvent accessible surface area
delta_unsatHbonds	Change in the number of unsatisfied hydrogen bonds upon complex formation
dslf_fa13	Energy from disulfide bond geometries
fa_atr	Attractive force between atoms from the Lennard-Jones potential
fa_dun	Internal energy of side chains based on statistics from Dunbrack
fa_elec	Columbic electrostatic force between atoms
fa_intra_rep	Repulsive force between atoms in the same residue in Lennard-Jones potential
fa_intra_sol_xover4	Lazaridis-Karplus implicit solvation energy within a residue
fa_rep	Repulsive force between atom from the Lennard- Jones potential
fa_sol	Lazaridis-Karplus implicit solvation energy
hbond_E_fraction	Fraction of dG_separated from interprotein hydrogen bonds
hbond_bb_sc	Hydrogen bond energy between backbones and side chains
hbond_lr_bb	Hydrogen bond energy between backbones of distant residues in primary sequence
hbond_sc	Hydrogen bond energy between side chains
hbond_sr_bb	Hydrogen bond energy between backbones of nearby residues in primary sequence
hbonds_int	Number of interprotein hydrogen bonds in the interface
lk_ball_wtd	Asymmetric Lazaridis-Karplus implicit solvation energy
nresl_all	Total number of amino acids in the complex
nres_int	Total number of amino acids in the interface
omega	Energy from omega dihedral angles
p_aa_pp	Probability of amino acid side chain conformations
packstat	Rosetta’s packing statistic score for the interface where 0 = bad and 1 = perfect
per_residue_energy_int	Average energy of interface residues
pro_close	Energy of proline ring closures
rama_prepro	Ramachandran preferences
ref	Reference energies for each amino acid
sc_value	How well the peptide and protein fit together
side1_normalized	The side1_score divided by the number of protein interface residues
side1_score	In PEPBI, the energy of the protein
side2_normalized	The side2_score divided by the number of peptide interface residues
side2_score	In PEPBI, the energy of the peptide
yhhplanarity	A special potential to keep TYR hydroxyls in the aromatic plane

While detailed descriptions of the binding property RIA outputs are available from Rosetta, brief descriptions are provided here to facilitate understanding of the PEPBI RIA binding property data.

## Data Records

PEPBI is freely available at https://datadryad.org/dataset/doi:10.5061/dryad.wstqjq2wk^51^. It consists of five primary components: (1) an Excel spreadsheet containing collected sequence, ITC, and RIA data for each protein-peptide complex entry, (2) a Python3 script that loads the Excel spreadsheet into a DataFrame and can generate a.csv file if desired, (3) the predicted peptide-protein complexes in PDB formatted files, (4) PDF files containing a visual representation of how the predicted complexes differ from one another and their source PDB file, and (5) a.csv file of the technical validation data.

The information in the spreadsheet is organized into three main sections: (1) the naming and sequence information for the peptide-protein complexes, (2) the ITC data, and (3) the RIA calculated properties. The first section begins with a “Binding Group” column, which identifies the protein (always first) and peptide (always second) of the complex. The names used in this column exactly match those from either the publication of the ITC data or the PDB file of the structure. This is followed by a “PEPBI Complex Name” column that reports the PEPBI identifier for the complex. It consists of abbreviated versions of the protein and peptide names as well as a numerical identifier of which variant from the ITC experiments the complex is. These three pieces of information are separated from one another by underscores. For example, fyn_p2l_1 is the PEPBI complex name for the first set of data in the Fyn SH3 – P2L binding group.

The third column in the first section of the spreadsheet is the “PDB ID” for the peptide-protein complex, which lists the accession code of the reference structure for the complex. This is followed by a “Crystallographic Unit” column, which identifies how many proteins (A) and how many peptides (B) are present in the PEPBI complex. For example, an entry of A2B2 would correspond to two copies each of the protein and peptide being in the complex. The next column is the “Unit Copy”, which lists the copy from the experimental crystallographic unit used to create the complex when multiple copies exist. These are listed as C1 for the first copy, C2 for the second, etc. This only occurs when a non-binary crystallographic unit has been decomposed into smaller units and “X” is used to indicate the absence of such information.

The next set of columns list information about the protein, including its name, its amino acid sequence using one letter amino acid codes, the number of amino acids in the protein, and any tags (e.g., poly-HIS) that were present on the protein during the ITC experiments. Such tags are included in the predicted complexes reported in PEPBI. The protein information is followed by similar information about the peptide, including its name, its amino acid sequence, and the number of amino acids in the peptide. Also included is which binding site on the protein the peptide interacts with, as some proteins had multiple peptide binding sites. This information is provided as “Site 1” or “Site 2” and only when the protein had multiple sites, with “X” used to indicate cases where proteins had only a single site. The identification of the binding locations in proteins with multiple sites is included in the files containing visual representations of the binding complexes. The final information about the peptide that is provided is the motif the protein recognizes, with “X” used to indicate cases where such information is unavailable. In the motifs, interchangeable residues at a single position are grouped within brackets and separated by slashes (e.g., [R/K]), repeated sequence units are indicated by brackets with an ‘n’ subscript (e.g., [R/K]n), and positions that can be any amino acid are represented by an “X”. An example motif would be [R/K]nX[R/K]W.

The final three columns in the first section of the spreadsheet detail how a particular PEPBI complex differs from the first complex in the binding group. This information is specifically listed in the “Change from First Binding Group Member” column, and options include changes to the peptide sequence, changes to the protein sequence, changes to the binding site of the peptide, changes to the temperature of the ITC experiments, and combinations of these. The first complex in a binding group has an “X” for this information, as there cannot be changes from itself. The next two columns list specific protein mutations and peptide mutations, respectively, when those are the changes. Single-site mutations are labelled by the original residue, its position in the sequence, and the new residue (e.g., P2G indicates the proline in position 2 is mutated to a glycine). Deletions are indicated in two ways: single-residue deletions include the original residue, its position, followed by ‘del’ to indicate deletion (e.g., S3del means the serine at position 3 is deleted), while multiple-residue deletions use a range, showing the first residue and its position and the last residue with its position separated by an underscore and trailed by ‘del’ (e.g., A1_A3del means the sequence from alanine at position 1 to alanine at position 3 is deleted). Insertions are denoted using ‘ins’, followed by the position where the insertion begins, then the residue(s) to be inserted. If multiple residues are added, the order of their placement follows the insertion position (e.g., ins13ASK means alanine is inserted at position 13, followed by serine at position 14, and lysine at position 15).

The second main section of the spreadsheet lists measured and calculated values from the ITC experiments. The first 13 columns in this section list values from the literature reports. This includes ΔG, k_D_, ΔH, TΔS, ΔS, and N (i.e., the stoichiometry of binding as defined in each original literature report). Standard deviations for these six values are included when they were reported, and the 13^th^ value is the T of the experiments. N is dimensionless, and the other properties use units involving kcal, mol, and K as appropriate. Values that were not experimentally reported are marked with an “X”. This is followed by three additional columns containing values of ΔG, TΔS, and ΔS calculated using the experimentally measured k_D_, T, and ΔH values.

The final main section of the spreadsheet includes the calculated RIA values for each peptide-protein complex. The 40 values are reported in the same order they are listed in Table 2.

The second main type of data in PEPBI are the predicted structures of the peptide-protein complexes in PDB-formatted files. These files are located in a “Binding Group Structures” folder, and each binding group has a subfolder for its complexes. The subfolders are labelled using the binding group names from the first column of the spreadsheet. In cases where there were multiple Crystallographic Unit classifications for a binding group, there is a subfolder for each such designation. In cases where there were different Unit Copies for a given Crystallographic Unit classification, there are further subfolders for each Unit Copy designation. Within the appropriate subfolder is a PDB-formatted file named using the PEPBI Complex Name of the peptide-protein complex (i.e., the label in the second column of the spreadsheet).

**Fig. 2:**
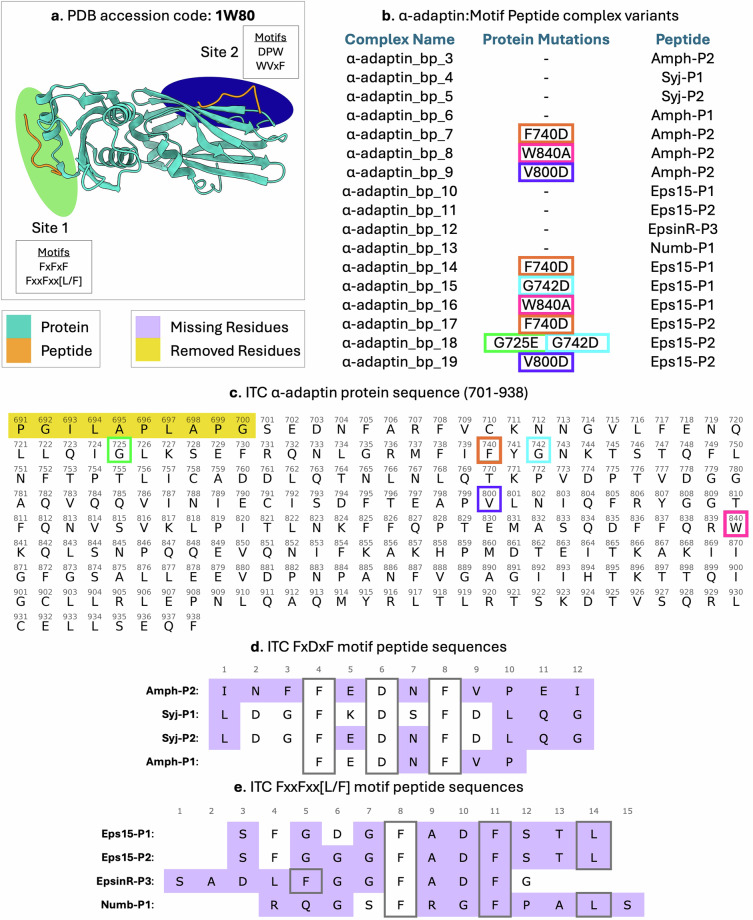
Structure Model Generation for α-adaptin:Motif Peptide (Site 1) Complexes. α-adaptin binds peptides at two sites. This image includes information for Site 1 only. Panel a is a visual depiction of the protein (teal) – peptide (orange) complex, including where the peptides bind to Site 1 of α-adaptin. Peptides with two motifs are known to bind α-adaptin at Site 1 and the motifs are listed in Panel a. Panel b lists the PEPBI complex names and the necessary adjustments to the structure for each complex. Positions in the protein that are mutated are color-coded in both Panel b and in the protein’s sequence in Panel c. Panels c-e show the protein and peptide sequences of the PEPBI complexes using one letter amino acid codes and include the numerical positions of the residues. The residue numbers match those in the PEPBI-predicted complexes as well as the original PDB file. Residues that were in the original, experimentally-determined structure but were not present during ITC experiments are highlighted in yellow. Such residues are not included in the PEPBI-predicted complexes. Residues that were different from those in the original, experimentally-determined structure are highlighted in purple. Such residues were mutated per the protocols described in the Methods.

The third major type of information PEPBI contains are summary files containing visual representations of the peptide-protein complexes made with UCSF ChimeraX^52^ version 1.7.1, how complexes that are part of the same binding group are related to one another, and how the experimentally determined structure in the PDB file was modified to predict the peptide-protein complexes. There is one such file per binding group and they are located in the subfolder of each binding group. The content of these files is best explained through example. Figure 2 illustrates the ‘α-adaptin - Motif Peptide’ binding group, focusing on members that bind to site 1. The figure includes a visual representation of the reference PDB structure used to model the binding group and a table detailing sequence alterations such as mutations, missing residues, or redundant residues. Lastly, the annotated protein/peptide sequences used in ITC studies are shown. Residues missing from the PDB file that were included in the ITC studies are highlighted in purple, while redundant residues not present in the ITC studies are highlighted in yellow. Additional mutations are color-coded between the table and sequences. The image also shows that site 1 binds two distinct motifs, FxDxF and FxxFxx[L/F], making the relationship between motif patterns and binding sites easily recognizable.

## Technical Validation

PEPBI aggregates previously reported ITC and protein structure experimental data. The existence of that data can be validated by checking the provided citations for the PepSet database, the ITC experimental reports, and the PDB files. The novel data in PEPBI consists of the predicted structures of the 329 peptide-protein complexes and the corresponding RIA-calculated values. As the structures analyzed by RIA are provided as part of PepSet, those values can be checked by independent use of RIA. The predicted complexes are each based on an identified PDB file and can be replicated by following the process described in the Methods section. Many freely available software programs (e.g., UCSF Chimera^45^) allow for the comparison of protein structures. Overall, the data in PEPBI can be validated either by checking prior literature reports or replicating the structure prediction process.

**Fig. 3:**
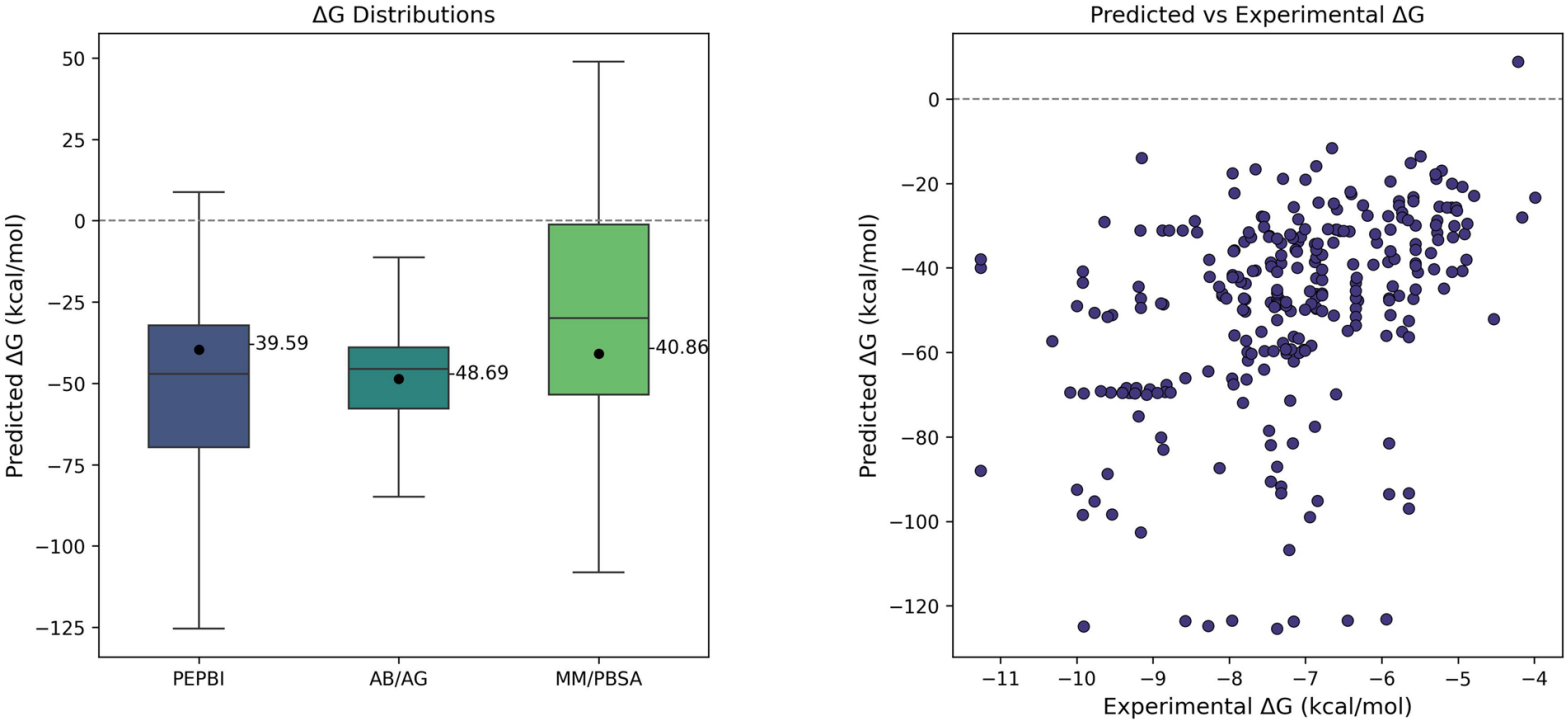
Comparison of Binding Energy Distributions and Correlation with Experimental Data. The left panel shows a box plot of predicted binding free energy (ΔG) distributions for three datasets. The PEPBI column shows the RIA-calculated binding energies in PEPBI. AB/AG applies the same prediction method to point mutants of antibody-antigen complexes, while MM/PBSA reports values calculated with a different method for protein-peptide systems. Black dots indicate mean values, and central lines represent the median values. Overall, the data demonstrates that the values in PEPBI are consistent with those from prior computational studies. The right panel presents a scatter plot comparing experimental ΔG values with their corresponding RIA predictions from PEPBI. For both plots, outliers outside the upper and lower limits of the predicted binding energies were excluded for clarity.

To validate the RIA-calculated binding free energies (ΔG) generated for the PEPBI database, the ΔG distributions were compared with two other studies: (1) 26,750 mutants of antigen-antibody complexes calculated using RIA^53^, the same method employed in PEPBI, and (2) ΔG values for 18 protein-peptide complexes predicted using the Molecular Mechanics Poisson-Boltzmann Surface Area (MM/PBSA) method^54^. Box plots, as shown in Fig. 3, were generated to visualize the predicted distributions of each dataset. The mean, median, and interquartile range (IQR) were calculated for each dataset to provide statistical context, where the IQR is the central 50% of each dataset. Outliers were excluded from visualization if they were more than 1.5 times the IQR above the 75^th^ percentile or less than 1.5 times the IQR below the 25^th^ percentile of the data. Across all datasets, the IQRs fell within the negative ΔG range, consistent with thermodynamic expectations for spontaneous and favorable binding. The two RIA datasets appear to be shifted somewhat more negative (i.e., favorable) than the MM/PBSA data, while the two peptide datasets appear to have a similar overall range to one another. Collectively, this suggests that the PEPBI ΔG values are consistent with what would be expected from prior computational studies.

The need for better methods of predicting ΔG values for peptides is illustrated in the second part of Fig. 3, which depicts the predicted values versus the experimental measurements of the 181 complexes in PEPBI. The plot shows minimal correlation between the values and demonstrates the inaccuracy of existing methods. The motivation for the development of PEPBI is to provide a dataset for model development that can yield better predictions.

## Data Availability

No custom codes were used to create the data in PEPBI, but a number of computational protein visualization and prediction tools were utilized. The specific versions of each were: UCSF Chimera version 1.17.1 for complex generation, UCSF ChimeraX version 1.7.1 for complex visualization, RoseTTAFold from the Robetta server (https://robetta.bakerlab.org), the REF15 parameterization of Rosetta, and CHARMM36.
